# Experimental validation of force inference in epithelia from cell to tissue scale

**DOI:** 10.1038/s41598-019-50690-3

**Published:** 2019-10-10

**Authors:** Weiyuan Kong, Olivier Loison, Pruthvi Chavadimane Shivakumar, Eunice HoYee Chan, Mehdi Saadaoui, Claudio Collinet, Pierre-François Lenne, Raphaël Clément

**Affiliations:** 1 Aix Marseille Univ, CNRS, IBDM, Turing Center for Living Systems, Marseille, France; 2 Department of Developmental and Stem Cell Biology, Institut Pasteur, 25 rue du Docteur Roux, 75724 Paris Cedex 15, France; 3 CNRS URA2578, rue du Dr Roux, 75015 Paris, France

**Keywords:** Biophysics, Morphogenesis

## Abstract

Morphogenesis relies on the active generation of forces, and the transmission of these forces to surrounding cells and tissues. Hence measuring forces directly in developing embryos is an essential task to study the mechanics of development. Among the experimental techniques that have emerged to measure forces in epithelial tissues, force inference is particularly appealing. Indeed it only requires a snapshot of the tissue, as it relies on the topology and geometry of cell contacts, assuming that forces are balanced at each vertex. However, establishing force inference as a reliable technique requires thorough validation in multiple conditions. Here we performed systematic comparisons of force inference with laser ablation experiments in four epithelial tissues from two animals, the fruit fly and the quail. We show that force inference accurately predicts single junction tension, tension patterns in stereotyped groups of cells, and tissue-scale stress patterns, in wild type and mutant conditions. We emphasize its ability to capture the distribution of forces at different scales from a single image, which gives it a critical advantage over perturbative techniques such as laser ablation. Overall, our results demonstrate that force inference is a reliable and efficient method to quantify the mechanical state of epithelia during morphogenesis, especially at larger scales when inferred tensions and pressures are binned into a coarse-grained stress tensor.

## Introduction

During embryonic development, a small set of coordinated cell behaviors, including cell division, cell death and cell shape changes, lead to dramatic changes in tissue shapes. These events rely on forces generated at the cell scale, which build up and induce tissue scale movements, such as tissue elongation, tissue invagination or tissue closure (reviewed in^1^). During epithelial morphogenesis, polarized contractile forces acting at cell junctions drive oriented cell intercalation and lead to convergent-extension^2–4^. Stress generated locally can also propagate passively within surrounding cells and tissues as in the *Drosophila* posterior midgut, which largely contributes to elongating the adjacent germband upon invagination^5,6^. The tight genetic control of force generation leads to remarkably stereotyped shape changes, which is exemplified by the robustness of morphogenesis at the embryo scale. A consequence is that misregulation of force generation patterns leads to important morphogenetic defects. Interestingly, such robustness can hold at the scale of a few cells, as revealed by the strikingly regular cellular arrangements of the *Drosophila* retina^7^.

A key step in understanding tissue morphogenesis is thus to establish reliable methods to assess the mechanical state of cells and tissues directly in the developing embryo. Evidently, measuring forces *in vivo* is not an easy task. A wide variety of techniques has recently been developed (for a review, see^8^), which include (but are not limited to) pipette aspiration^9,10^, magnetic tweezers^11^, laser cuts^3,12^, photoelasticity^13^, or deformable microdroplets^14^. All these techniques require to access the tissue of interest with a probe, and are therefore invasive and technically challenging. Optical tweezers have been used to perform non-invasive mechanical measurements at single junctions^15,16^, yet they only provide a small number of local measurements per embryo, and are thus difficult to implement to map the distribution of forces within a tissue. Force inference, which relies on the hypothesis that tensions equilibrate at each vertex, uses the geometry of cell contacts to infer a map of tensions and pressures from a tissue image^17–20^. Because force inference is non-invasive and does not require a specific experimental setup, it stands out as a simple and convenient method.

As pointed out in a recent review^8^, it is now crucial to cross-validate different measurement techniques in model systems in order to assess their robustness and reliability. Such cross-validation experiments require the combination of two or more techniques, and each of them being a technical challenge, cross-validation efforts remain rare in this rather new field of research.

Here, we investigate the accuracy of force inference using cross-validation with laser ablation experiments. Ishihara and co-workers combined force inference and annular laser cuts to show that force inference could predict coarse stress polarity averaged over the whole field of view in the *Drosophila* notum^21^. However, a systematic, detailed cross-validation of force inference in different conditions and at different scales is chiefly missing, in particular for complex tension and stress patterns. To that end, we carried out our analysis at various spatial scales, in four distinct epithelia from two different animals, the fruit fly and the quail. We first validate our force inference algorithms on synthetic data. We then turn to the *Drosophila* notum, and study single junction tension, showing that force inference correlates fairly well with the recoil velocity of vertices following junctional laser cuts. We next turn to the *Drosophila* retinal ommatidia, and show that force inference adequately predicts tension patterns in these stereotyped groups of cells, in both wild type and mutant conditions. Finally, we show that force inference can predict complex tissue-scale stress patterns with unprecedented precision in the wild type and mutant *Drosophila* germband and in the quail early embryo.

Altogether, our cross-validation study on different tissues demonstrates that force inference can be confidently used in 2D to assess the mechanical state of a variety of epithelial tissues. As accuracy increases with the level of coarse graining, we believe it is particularly well suited to determine complex stress patterns at the tissue scale during morphogenesis.

## Results

### Preliminary – choice and validation of the inference methods

Force inference is an inverse problem of mechanics, which aims at inferring the tensions and pressures that cause angle variations at cell vertices by precisely measuring these angles. It thus requires writing force balance equations at each vertex. A general difficulty is the indefiniteness caused by image boundaries, where edges are connected to one vertex only. The full inverse problem (one tension per edge and one pressure per cell) is indeed generally underdetermined, with fewer equations than unknowns^21^. Different strategies can be adopted to handle this indefiniteness and yield a plausible set of tensions and pressures. First, one can assume that intracellular pressure is constant across the tissue. The problem then becomes overdetermined (more equations than unknowns) and can be solved by computing the pseudo-inverse of the associated matrix^18^. As edges are rarely perfectly straight, suggesting pressure differences between cells, we chose to discard that assumption. Second, one can complement the contact angles measurements with the measurement of the radii of curvature between each pair of adjacent cells. Using Young-Laplace law, this provides another set of conditions that again lead to an overdetermined problem^20^. This is an ideal solution if edge tangents (for tensions) and curvatures (for pressures) can be accurately measured. Third, one can adopt a Bayesian approach, and incorporate statistical expectations for the system as a prior, for instance assuming a Gaussian distribution of tensions^19^. This is a good strategy when curvature measurements are difficult. In all cases, force inference provides relative tension estimates (and so do ablation experiments), as they are determined up to a multiplicative constant. Inferred pressures are determined up to an additive constant (hydrostatic pressure). A common convention is to scale tensions so that the average tension is 1, and to fix the reference average pressure to 0.

Before applying force inference to biological tissues, we first used data generated in silico, a standard procedure to validate proper implementation^18–20^. We generated synthetic data using Surface Evolver^22^, a software that uses energy minimization to drive a system governed by custom line/surface energies to equilibrium (see methods). Briefly, known tensions and pressures are assigned to a regular array, which is then driven to equilibrium. This allows direct comparison of inferred tensions and pressures to known tensions and pressures. Bayesian inference performs very well, as shown by comparisons between the true versus inferred tension and pressure maps (Fig. S1A–C). The correlation is excellent for both tension and pressure, with a Pearson’s correlation coefficient above 0.9 (Fig. S1D,E), as expected for synthetic data^19^. We used a similar validation approach to validate our Laplace inference code. This time we used simulations of groups of cells mimicking *Drosophila* ommatidia, the only experimental system that we analyze with Laplace inference (see below). Again, we find an excellent agreement between simulations and force inference, as shown by the tension and pressure maps (Fig. S1F–H). Although the system only has 6 cells and 13 edges, the correlation remains excellent for both tensions and pressures (Fig. S1I,J).

In this article, we preferentially used Bayesian inference^19^ for tissues with a large number of cells and small curvatures, that is, the *Drosophila* notum and germband, and the quail embryo. Indeed, we noticed that Laplace inference^20^ is prone to error propagation when the system size increases (see methods and Fig. S2 for details). Briefly, this is due to the difficulty to properly determine edge tangents at vertices. Measuring tangents and curvatures requires fitting segmented, pixelated edges. This procedure can introduce errors that propagate to neighboring vertices and edges when the inverse problem is solved. This effect is substantial in the tissues mentioned above, as curvatures are usually tiny and thus hard to determine. Besides, edges often appear as a very open S upon segmentation. This is a typical source of dramatic projection errors upon tangent determination. In contrast, Laplace inference is very well suited for the ommatidia of the *Drosophila* retina. Indeed, ommatidia are stereotyped units composed of only 6 cells with highly stereotyped shapes and very high curvatures, which allow averaging and therefore much easier and reliable measurements of tangents and curvatures.

### Single junction tensions in the *Drosophila* notum

**Figure 1:**
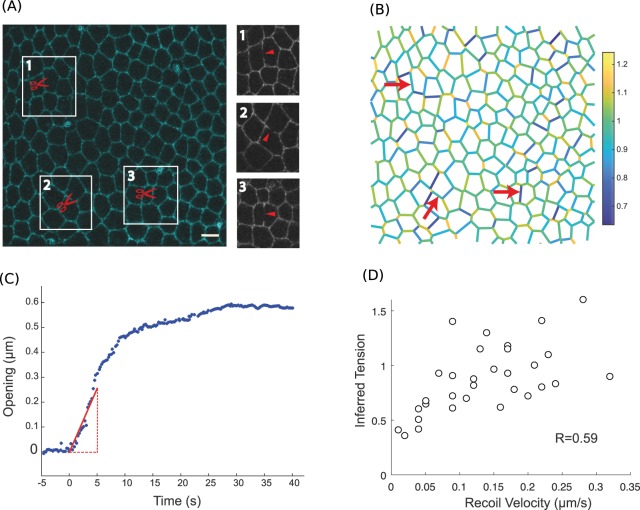
Force inference at the single junction scale in the Drosophila notum. (**A**) Subregion of the Drosophila notum 21 h after pupa formation. Scissors show ablation spots where recoil velocities will be measured. Insets show post-ablation snapshots of the considered junctions. Scale bar: 5 μm. (**B**) Inferred tension map of the tissue region in (A) before the ablations. Red arrows indicate the location of ablations, where inferred tensions are extracted and compared to experimental recoil velocities. (**C**) Opening dynamics and initial recoil velocity. The red line shows a linear fit of the first 5 seconds, which is used to determine the initial recoil velocity. (**D**) Inferred tension vs. opening velocity (N = 31 laser cuts from 10 pupae). Pearson’s correlation coefficient is 0.59. Spearman’s correlation coefficient is 0.63.

The most straightforward experimental verification of force inference accuracy is to directly compare tensions inferred in single junctions to measurements obtained from single junction laser ablation, which is the most common experimental technique to evaluate junction tensions. In laser ablation experiments, a tightly focused laser disrupts the molecular structures that support tension in a targeted junction. Upon release, tension is only balanced by fluid friction, so that the opening velocity following ablation is proportional to tension^23^. Providing that friction is the same among cuts, ablation thus provides relative estimates of tension. To compare force inference to laser ablation in single junctions, we used a rather regularly organized epithelium, the pupal notum of *Drosophila* around 21 h after pupa formation (Fig. 1A). Tension variations at this stage are not expected to be particularly oriented, as revealed by annular laser cuts^24^. Hence they are essentially random fluctuations that cause the system to slightly deviate from a regular array. Because force inference provides relative estimates, it is always delicate to compare tensions estimated from separate images. We thus hypothesized that the average tension was always the same in all of our images (normalized to 1). To moderate the influence of this assumption, for each field of view where force inference was performed, we did several (3 to 5) laser cuts, sufficiently spaced so as not to influence each other (Figs 1A, S3). Force inference was computed in an image taken prior to the laser cuts (Fig. 1B). We compared the inferred tensions to the initial recoil velocities of the cut junctions, measured by fitting the onset of the opening (Fig. 1C). We found a fairly good correlation coefficient of about 0.6 between opening velocities and inferred tensions (Fig. 1D). The discrepancy can arise from numerous sources: the intrinsic hypotheses of force inference, but also the errors made on velocity measurements, and the assumptions that tension is solely balanced by pure fluid friction and that fluid friction is homogeneous in the tissue. The correlation found despite these limiting factors suggests that both methods can provide reliable results. Of note, the ratios between the recoil velocities are not the same as the ratios between inferred tensions. This is not the case in simulations, which suggests that the error might arise from laser ablation experiments. Besides experimental noise, this might result from systematic nonlinear friction effects (friction force not simply proportional to velocity, so that the recoil velocity is not simply proportional to tension). In addition, since recoil velocities are estimated from a linear fit at the onset of the opening, an error is clearly made by approximating relaxation by a linear fit. The error made actually depends on the relaxation timescale, and thus on tension, which could also be a systematic source of error.

Clearly, measurements in single junctions are overall likely to be prone to more errors than measurements averaged over groups of junctions. Such groups can be based on position (coarse graining), orientation (to detect polarity), or biological identity. We therefore questioned next whether force inference could detect tension gradations between different, stereotyped groups of junctions.

### Tension patterns in wild type and mutant *Drosophila* ommatidia

**Figure 2:**
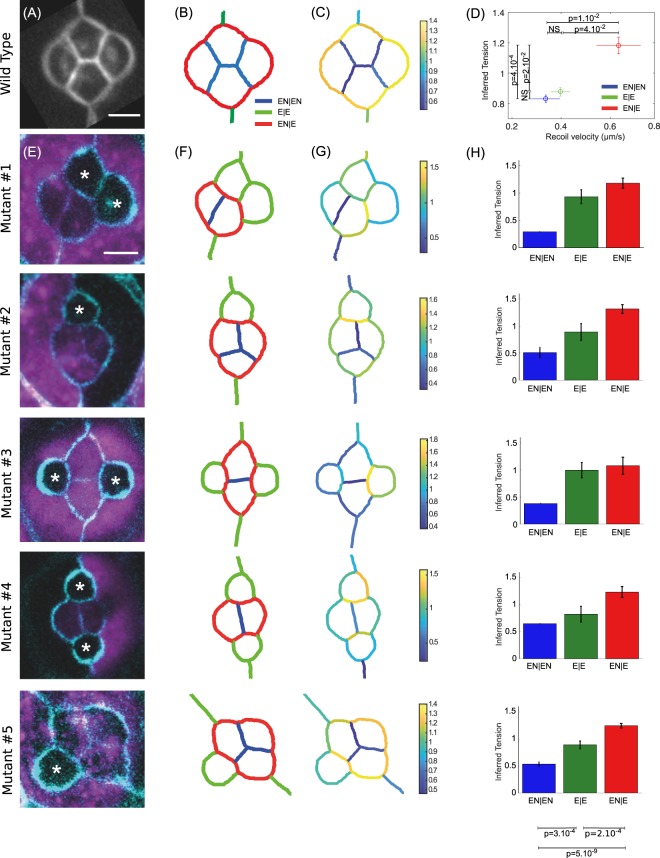
Force inference in the Drosophila retina. (**A**) The four cone cells of a WT ommatidium. The image results from an average over N = 51 ommatidia. Scale bar: 5 μm. (**B**) Segmented version of (**A**), and nomenclature of the junction types: EN|EN junctions in blue, E|E in green, and EN|E in red. (**C**) Map of inferred tensions. (**D**) Mean inferred tension vs. mean recoil velocity for each junction type (EN|EN: N = 19, E|E: N = 16, EN|E: N = 22). (**E**) Five different mutant configurations generated from the mosaic experiments. WT cells are in purple. Starred cells do not express N-Cad. This only affects cone cells, as surrounding cells do not express N-Cad. Scale bar: 5 μm. (**F**) Pattern of junction types for each configuration. (**G**) Map of inferred tension in a single ommatidium for each configuration. (**H**) Average inferred tension for each junction type in each configuration (statistical tests at the bottom pull the five mutant configurations together).

Stereotyped patterns of differential tension between subgroups of cells can drive robust geometric organization of multicellular structures. We wanted to assess whether force inference could detect such patterns of tensions. To achieve this, we turned to the retina of *Drosophila*, composed of highly stereotyped groups of cells called ommatidia (Fig. 2A). Previous studies showed that cone cell shapes and arrangement in ommatidia are determined by stereotyped differential tensions^7,25–27^. These tensions were shown to be determined by the amounts of Myosin-II (Myo-II) and E- and N- cadherins recruited at the considered junctions^27^. These amounts were in turn shown to be determined by the “identity” of junctions, that is, by the types of cadherins expressed in the two contacting cells^27^. Based on these previous results, we categorized junctions according to the cadherins expressed in the contacting cells. EN|EN junctions correspond to homotypic junctions separating two cells that both express E- and N-Cadherin. E|E junctions correspond to homotypic junctions separating two cells that both express E-Cadherin only. EN|E junctions correspond to heterotypic junctions separating a cell expressing E-Cadherin only from a cell expressing both E and N-Cadherin. These three types of junctions coexist in a wild type ommatidium (Fig. 2B).

We computed the averaged opening dynamics following laser cuts for each type of junctions, and extracted the corresponding initial recoil velocity (Fig. S4). As previously demonstrated^27^, this revealed a gradation of tensions according to junction type. Homotypic EN|EN junctions have the lowest tensions, E|E junctions have intermediate tensions, and heterotypic EN|E junctions have the highest tensions (Fig. 2D). Note that tensions are directly related to the amounts of Myo-II present at these junctions^27^. To perform force inference in this system, we averaged the geometry of N = 51 ommatidia, and segmented the resulting image (Fig. 2C). As stated earlier, high and stereotyped curvatures in this system make it possible to properly measure the tangents and radii of curvature required for Laplace inference. We found that Laplace inference accurately predicts the pattern of tensions and its gradation among the three types of junctions (Fig. 2D). Note that the cell pressures can also be computed. As expected from Young-Laplace law, the pressure is higher in cone cells than in the surrounding cells (Fig. S5A).

We then turned to the analysis of mosaic experiments in which a fraction of cells do not express N-Cadherin^27^. Since the mutation affects random cells in the tissue, such experiments generate a variety of configurations, in which one or more cone cells are affected by the mutation (Fig. 2E). Interestingly, this modifies the pattern of junction types in the ommatidia, since junction type is determined by which cadherins are expressed by the contacting cells (Fig. 2F). To test whether force inference could still detect tension gradation in these modified conditions, we applied force inference to 5 different configurations of ommatidia (Fig. 2G). Note that, due to the stochastic generation of these configurations, inference is performed on a single ommatidium for each configuration, whereas an average over many ommatidia was used for the wild type condition. Strikingly, the gradation of tensions identified in the wild type condition is systematically detected by force inference in the various mutant configurations (Fig. 2H). This suggests that tensions are indeed determined by the combination of cadherins expressed by adjacent cells, through adhesion strength but also Myo-II level^27^. Inference results are also consistent with laser cuts averaged over all mutant configurations for each junction type (Fig. S5B).

Overall, the results obtained in the retina suggest that force inference can robustly detect tension patterns in stereotyped units of a few cells. This led us to investigate the ability of this technique to detect stress patterns at the scale of the tissue, relevant to many morphogenetic processes.

### Stress pattern in the avian embryo

**Figure 3:**
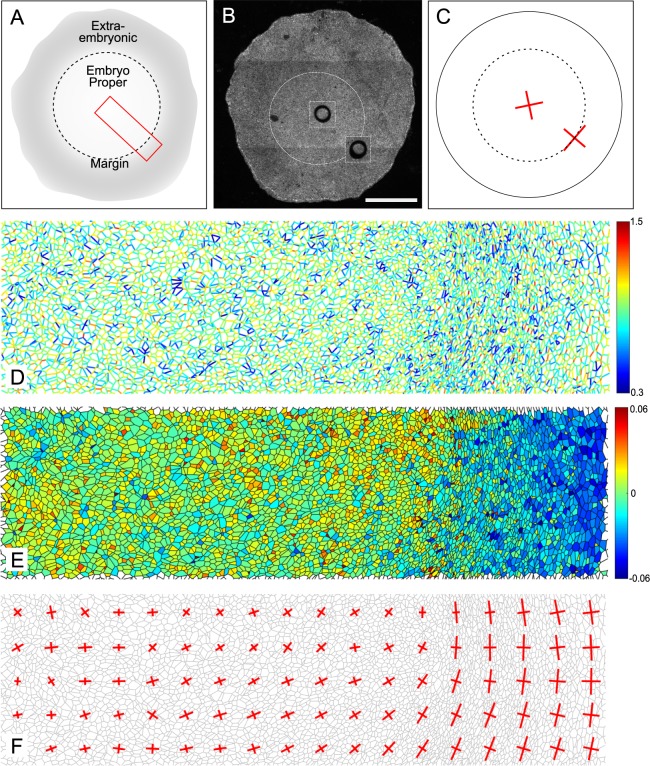
Tissue-scale force inference in the quail embryo. (**A**) Schematics of the embryonic and extra-embryonic territories. The red box shows the radial region analyzed with force inference. (**B**) Typical regions used for ablations in the embryonic region and in the posterior margin region. Images 2 minutes after a cut are superimposed on the original image. Scale bar: 1 mm (**C**) Strain measured in the embryonic and margin regions 2 minutes after the cut (N = 7 from 4 embryos). Red crosses show the principal directions and amplitudes of tissue strain measured 2 minutes after the cuts. (**D**) Map of inferred tensions. (**E**) Map of inferred pressures. (**F**) Map of inferred stress. Red crosses show the principal directions and amplitudes of the stress tensor.

Force inference, by coarse graining tensions and pressures at the appropriate scale, can be used to build a map of the stress tensor^19,28^. Very promising results were obtained using this approach to determine the complex stress pattern of the entire *Drosophila* notum^29^. However comparison to experimental stress measurements was only performed at a very coarse level, looking at the overall anisotropy of the whole field of view, by binning tensions and pressures on the whole sample^21^. To investigate the ability of force inference to detect complex stress patterns at large scales, we first turned to the gastrulating avian embryo, using the quail as a model system (Fig. 3A). Although *Drosophila* is the most common model animal to study epithelial mechanics and epithelial morphogenesis, there is no reason that force inference general principles should not apply to other animals. At this early stage, the quail primitive ectoderm is essentially flat with about 10^5^ cells. Previous studies carried out in chicken and quail during gastrulation have shown the presence of tangential Myosin cables at the margin between the embryo proper and the extra-embryonic territory, driving convergent extension of the presumptive primitive streak^30,31^. To test our force inference in this system, we used circular laser cuts and segmentation of fixed samples stained for ZO-1 (Fig. 3B), which labels the apical membrane, as described in^31^. As previously reported, the deformation following laser cuts is isotropic within the embryo proper, suggesting isotropic stress, but anisotropic at the margin. The principal direction of stress at the margin is orthoradial, that is, tangential to the margin itself (Fig. 3C). Due to the very large number of cells in the whole embryo, we restricted force inference to a region spanning radially from the center of the embryo to the posterior margin (red box, Fig. 3A). We did not detect obvious patterns of junctional tension amplitude (Fig. 3D). We then computed a coarse-grained stress tensor, obtained by binning the results of force inference over square subregions of few tens of cells (see methods). We then plotted its principal directions and amplitudes in each subregion, which recapitulates the anisotropy gradient revealed by circular ablations (Fig. 3F). In the embryonic territory, we find no stress anisotropy. As we get closer to the margin, stress gradually becomes anisotropic and oriented along the orthoradial direction, consistent with the outcome of circular laser cuts. Interestingly, we also observe a pressure gradient across the tissue, with higher pressures in the embryonic territory (Fig. 3E), which might be indicative of differences of mechanical state between the embryonic and extraembryonic territories.

This last analysis confirms the ability of force inference to detect stress patterns at the scale of thousands of cells, and shows that the approach is relevant to animals other than *Drosophila*. However, due to the very large size of the system and to experimental limitations preventing from directly measuring recoil velocities (see methods), our analysis remains essentially qualitative. This prompted us to perform another set of experiments in a system amenable to more precise quantifications.

### Stress pattern in the wild type and mutant *Drosophila* germband

To that end, we turned to a mechanically well-characterized tissue, the embryonic germband of *Drosophila*, that is known to display stress polarity induced by Myo-II polarity, but also a stress gradient along the antero-posterior (AP) axis, due to the movement of the posterior midgut pulling on the tissue^5,6^.

**Figure 4:**
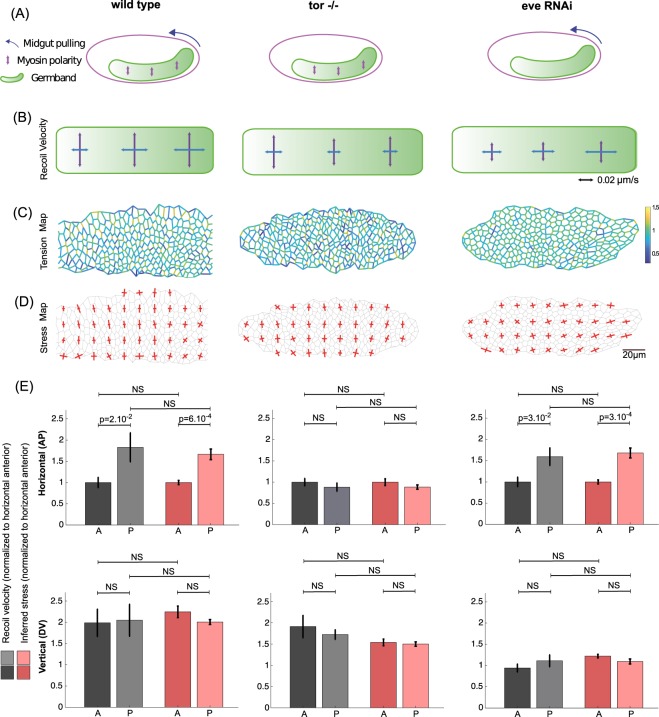
Tissue scale force inference in the Drosophila germband. (**A**) Scheme of stress sources in the germband. In the WT condition (left), Myo-II polarity generates stress along the DV axis, and posterior midgut invagination pulls on the germband from its posterior side. In the Tor−/− condition (middle), posterior midgut invagination is abolished, and Myo-II polarity is preserved. In the Eve RNAi condition (right), posterior midgut invagination is preserved, and Myo-II polarity is abolished. (**B**) Recoil velocities measured with PIV for each condition in the anterior, middle and posterior regions of the germband. Vertical arrows correspond to opening velocities along the DV axis (cuts along the AP axis), and horizontal arrows correspond to opening velocities along the AP axis (cuts along the DV axis). Each arrow results from an average over N = 7 to N = 34 experiments. (**C**) Map of inferred tension, in a representative germband for each condition. (**D**) Map of inferred stress. Red crosses show the principal directions and amplitudes of the stress tensor. (**E**) Bar plots of normalized recoil velocity and inferred stress in the horizontal direction (top row) and in the vertical direction (bottom row) for each condition. A stands for anterior, P for posterior. Anterior (resp. posterior) inferred stress is computed as an average over the three most anterior (resp. posterior) columns of (**D**).

In the wild type germband, the polarized recruitment of Myo-II at dorso-ventral (DV) junctions is known to polarize stress and induce polarized cell intercalation. In addition, the posterior midgut, which undergoes rotation and invagination, has been shown to pull on the germband along the AP axis, inducing an additional gradient of stress along this axis (Fig. 4A, left panel). This is illustrated by the opening velocities measured following large AP- or DV-oriented line cuts performed in the anterior, middle and posterior regions of the germband (Fig. 4B, left panel). In the anterior region, away from the posterior midgut, stress is dominated by Myo-II polarity and is strongly polarized along the DV axis. In the middle region, getting closer to the pulling posterior midgut, stress along the AP axis increases, but remains smaller than stress along the DV axis. In the posterior region, stress along the AP axis becomes even larger due to the proximity to the posterior midgut, and stress along the AP and DV axes become comparable, so that stress polarity is lost. We performed force inference on the germband during this process. First, the tension map shows that tensions are indeed higher along the DV axis than along the AP axis (Fig. 4C, left panel), as abundantly reported in literature^3,15,32^. To obtain a better representation of polarity, we computed the stress tensor, binning over square subregions of typically 8–10 cells. We then plotted its principal directions and amplitudes in each subregion (Fig. 4D, left panel). The results are fully consistent with the laser cut experiments. In the anterior region, stress is largely polarized along the DV axis. Getting closer to the posterior, stress along the AP axis gradually increases, so that in the posterior region, DV polarity is strongly reduced. To further quantify the stress gradients, stress polarity, and the agreement between laser cuts and stress inference, we averaged inferred AP and DV stress in the anterior and posterior regions, and directly compared them to the measured AP and DV recoil velocities in these regions (Fig. 4E, left panels). We find an excellent quantitative agreement between inference and laser cuts. Note that this is also exemplified by a plot of stress anisotropy along the AP axis (Fig. S6). To further test force inference ability to detect stress patterns, we used mutant conditions in which posterior midgut invagination (Torso−/−) or Myo-II polarity (Eve RNAi) are selectively impaired. In the absence of posterior midgut invagination, posterior pulling forces are abolished (Fig. 4A, middle panel), and the stress pattern is mostly determined by Myo-II polarity, with an important DV stress polarity from anterior to posterior (Fig. 4B, middle panel). This is fully recapitulated by the force inference approach (Fig. 4C–E middle panels). In contrast, in the absence of Myo-II polarization, the stress pattern is mostly determined by the posterior forces (Fig. 4A, right panel). Laser cuts show that stress along the DV axis is reduced across all the tissue, while the gradual increase of stress along the AP axis from anterior to posterior is maintained (Fig. 4B, right panel). The stress pattern is again fully recapitulated by the force inference approach (Fig. 4C–E right panels).

Altogether, the analyses of normal and impaired germband extension show that force inference can precisely recapitulate complex stress patterns across a dynamic epithelium undergoing morphogenetic movements. Force inference is also much faster than laser ablations, and the spatial resolution of the estimated stress is much higher.

### Stress tensor based on cell shapes only

Last, we questioned whether cell shape anisotropy alone was a good indicator of stress anisotropy predicted by force inference at the tissue scale. Interestingly, an approximate stress tensor can be computed without solving the inverse problem, if one assumes that all tensions and pressures are homogeneous. This yields an approximation that is solely based on the contribution of cell shape anisotropies detected by segmentation. Indeed, junction orientations contribute to the stress tensor, with tensions being prefactors (see Batchelor’s formula in the methods). Thus, the anisotropy of junction orientation, regardless of tensions, contributes to the anisotropy of stress. Similarly, gradients of junction length will contribute to gradients of stress magnitude.

This simplified analysis actually provides a good approximation of the stress obtained by force inference at the tissue scale, suggesting that junction orientation statistics largely contributes to determining the stress tensor. In the *Drosophila* germband, the error is negligible in most subregions (Fig. S7), even though junction tensions themselves are anisotropic (Fig. 4C). In the quail embryo, this approximation still yields good results, although the error is more important, especially close to the margin (Fig. S8). Besides this deviation from the result obtained with force inference, an obvious drawback of this simplified approach is that it cannot detect junction tension polarity or cell pressure gradients. However, an important advantage is that it does not require to implement force inference, but only segmentation and elementary computation. It is also much faster than force inference, especially in large systems where solving the inverse problem becomes computationally demanding. Finally, the results obtained with this approach suggest that even segmentation might not be mandatory, if the cell shape anisotropies can be properly detected from appropriate spectral analysis of the tissue image. This strategy was recently used to determine stress using Fourier transforms^33^. Our results suggest that it is certainly an interesting option, especially for tissues with a very large number of cells, or tissues in which segmentation is challenging due to imaging difficulties.

## Discussion

Advantages of force inference include that it is fully non-invasive, much easier to perform than perturbative experimental measurements, and does not require assumptions on the origin of forces involved or on the tissue rheology. However, it also comes with several assumptions. First and foremost, that tissue mechanics is essentially driven by in plane tensions and pressures. Second, that tensions are positive, and constant along cell edges. Third, that tensions equilibrate at each vertex, in other words that the magnitude of the net force at vertices (and thus of the friction force opposing movement) should be small compared to individual junctional tensions. This assumption is experimentally verified in various epithelial tissues, the velocity of cells and vertices during development being much smaller than the recoil velocity upon junction ablation. However, it is important to say that tissues in which edges are not tensed (wiggly junctions), tissues that significantly deviate from a 2D plane, or very dynamic tissues, should be considered more carefully. Whether force inference could confidently be used under these assumptions in classic epithelial monolayers was still unclear. Here, we conducted a thorough cross-validation in various epithelial tissues chosen from two animals, at different developmental stages, and with different geometries and dynamics. Our results demonstrate that force inference can be reliably used to analyze the mechanical state of various epithelia, from a few cells to thousands of cells.

We showed that force inference allows fairly good estimates of tension at single junctions. By providing a large set of measurements from single images, force inference can therefore be an asset to search for correlations between tension and protein distribution with good statistics^32^. However, we demonstrated that averaging over groups of junctions of interest, or coarse-graining tension and pressure into a binned stress tensor, significantly improved the reliability of the pattern detected. This is especially striking at the tissue scale. For the germband, a dynamic, morphogenetic tissue with a complex stress pattern, the advantages of using inference were obvious compared to laser cuts, which can be painfully long to perform as they require averaging over many animals. Inference over a single, well-segmented germband not only recapitulated the ablation findings but also allowed a more precise characterization of the stress pattern. Moreover, results obtained in the quail show that such tissue-scale analyses are robust in animals other than *Drosophila*. Taken together, our analyses of the *Drosophila* germband and of the quail embryo show that force inference is particularly well suited to determine stress patterns at the tissue scale during morphogenetic events, as previously done by Guirao and coworkers^29^. Considering that cell movements are likely to induce friction, this suggests that it remains small enough that the hypothesis of equilibrium at vertices remains valid. Force inference could also be an asset to study stress propagation and tissue rheology during morphogenesis, as tissue- or even animal-scale stress patterns and tissue flows can be established by active forces generated locally^34^.

## Methods

### Segmentation

We used the Tissue Analyzer plugin for FIJI to segment our images^35^. The segmented data (vertices, edges, connectivity) was then passed to Matlab and used for force inference.

### Bayesian force inference

Bayesian force inference was implemented in a custom Matlab script. The mathematical formulation of the method was first introduced by Ishihara and coworkers^19^. Both tensions and pressures are written as forces acting directly on vertices. The curvature of edges is not considered to solve the inverse problem. Tensions and pressures are determined simultaneously, and the problem is therefore underdetermined. A Gaussian prior on tension distribution is used to overcome the underdetermination. We used SuiteSparse to perform QR decomposition in Matlab^36^.

The stress tensor (Figs 3F and 4D) is then determined using Batchelor’s formula^19,28^:

$${\sigma }_{\mu \nu }=(-\sum _{i=cells}\,{P}_{i}{a}_{i}{\delta }_{\mu \nu }+\sum _{[ij]=edges}\,{T}_{ij}\frac{{l}_{ij}^{\mu }{l}_{ij}^{\nu }}{||{{\boldsymbol{l}}}_{ij}||})/\sum _{i=cells}\,{a}_{i}$$

where *a*_*i*_ is the area of cell *i*, *P*_*i*_ its pressure, *δ* is Kronecker’s symbol, *T*_*ij*_ is the tension of the edge [*ij*] separating cells *i* and *j*, and ***l***_***ij***_ the vector connecting the two vertices of edge [*ij*]. Red bars show principal directions of *σ*, and their length is proportional to the corresponding eigenvalues. Note that it is up to the user to choose the appropriate level of coarse-graining. Stress can be computed separately for each cell, or averaged over subregions of any desired size (here, 8–10 cells in the germband, and few tens of cells in the quail embryo).

### Laplace force inference

Laplace force inference was implemented in a custom Matlab script. The mathematical formulation of the method was introduced by Broadland and coworkers^20^. First, tensions are determined separately by measuring the tangents at each vertex and solving force balance for the whole system (which is then independent of pressures and thus overdetermined). To determine the tangents, we performed linear fits of the first pixels of each edge (8 pixels in our analysis of ommatidia). Next, the curvature of each junction is measured using Taubin circle fitting method^37^. Once curvatures are determined, pressures can be computed using Laplace’s law for each pair of adjacent cells. Again, this is an overdetermined system. Note that pressure determination is not crucial to our analysis of ommatidia, since we have no experimental data concerning cell pressures.

To test Laplace force inference with Surface Evolver, we first compute the equilibrium geometry with Surface Evolver, then compute a segmented mask image that we pass to Matlab for analysis. We used a default resolution of 50 pixels/edge for the mask images (on average, as edges have variable lengths). Note that the resolution affects the determination of tangents and curvatures, and thus the accuracy of force inference.

As stated earlier, Laplace force inference is ideal if tangents and curvatures can be determined accurately. Unfortunately, most of the time this is not the case. Indeed, in tissues such as the notum, the germband, the quail embryo or any other similarly organized monolayer, curvatures are small. This can make tangent and curvature measurements tough because of edge pixelation. Even more so, imaging and/or segmentation limitations can result in edges in the shape of a very open S (Fig. S2A), which not only makes tangents and curvatures difficult to assess but can also generate non-compatible angles for the two tangents at both extremities of an edge. Consequently, this inference method is very sensitive to tangent determination through fits of the edges first pixels, and to the number of pixels chosen for the fit. Using too few or too many fit pixels dramatically affects the result of Laplace force inference (Fig. S2B). We also find that determining tangents with circle fits of the whole edge (instead of linear fits of the first few pixels) yields poor results. Errors made on tangents propagate to neighboring edges when the matrix is inverted, which can lead to artefactual gradients in the inferred tension maps (Fig. S2B). A consequence of error propagation is that larger systems are prone to more error (Fig. S2C). This is true for our code, but even more so for CellFIT^20^. Note that we kept the resolution constant when we changed the system size (~50 px/edge). To further investigate error propagation, we introduced errors in a given simulation’s inference matrix. First, we computed the matrix of a simulated tissue. We introduced a random error (up to 10%) on a single non-zero projection coefficient randomly chosen in the matrix. We then solved the inverse problem, and computed the correlation between the inferred tensions with and without error introduction. Repeating this operation hundreds of times shows that introduction of an error on projection angles rapidly affects the overall result, even more so when the system has more cells (Fig. S2D). Finally, we provide as examples of error propagation the tension maps provided by our code and by CellFIT for a wild-type *Drosophila* germband (Fig. S2E). Clearly, these maps have artefactual tension gradients, that are not present when we use Bayesian inference. In contrast, Laplace inference is very well suited for ommatidia, as they have high curvatures and only few cells.

### Tissue simulations (surface evolver)

The synthetic tissue data was generated using Surface Evolver v2.7^22^. Surface Evolver evolves the given surface towards its minimal energy configuration by a gradient descent method. In our case we used a classical energy function of the form^38,39^:

$$E=\sum _{[ij]=edges}\,{\gamma }_{ij}{l}_{ij}+\frac{1}{2}\sum _{i=cells}\,{k}_{p}{({p}_{i}-{p}_{i}^{0})}^{2}+\frac{1}{2}\sum _{i=cells}\,{k}_{a}{({a}_{i}-{a}_{i}^{0})}^{2}$$

where *p*_*i*_ and *a*_*i*_ are the perimeter and area of cell *i*, and ${p}_{i}^{0}$ and ${a}_{i}^{0}$ its target perimeter and target area. *k*_*p*_ and *k*_*a*_ are the strengths associated to the perimeter and area constraints, respectively. *γ*_*ij*_ is the line tension in edge [*ij*], and *l*_*ij*_ its length.

The pressure in cell *i* (“known” pressure) is then given by ${P}_{i}=-\,{k}_{a}\,({a}_{i}-{a}_{i}^{0})$, and the total tension of edge [*ij*] (“known” tension) is given by ${T}_{ij}={\gamma }_{ij}+{k}_{p}({p}_{i}-{p}_{i}^{0})+{k}_{p}({p}_{j}-{p}_{j}^{0})$.

In the tissue simulation (Fig. S1B), the target area is set to 0.87 and *k*_*a*_ is set to 2. For the sake of simplicity, the target perimeters are all set to 0. *k*_*p*_ is set to 0.15. Line tensions *γ*_*ij*_ are randomly assigned from a Gaussian distribution (mean = 1, std = 1/6) prior to equilibration.

In the ommatidium simulation (Fig. S1G), the target area is set to 5.5 for top-bottom cone cells, 5 for right-left cone cells and 40.49 for surrounding cells. *k*_*a*_ is set to 38.2 and *k*_*p*_ is set to 0. Tensions *T*_*ij*_ = *γ*_*ij*_ are directly set to 0.75 for E|E junctions, 0.6 for EN|EN junctions, and 1.45 for EN|E junctions.

Note that whether these values are realistic or not is of little concern, since their only purpose is to demonstrate the proper implementation of force inference.

### Tissue curvature

Most biological tissues are not completely flat. When using force inference, one should be careful about this aspect, as approximating out-of-plane forces looking at their 2D projection can introduce significant errors if the curvature is important. For the tissues analyzed in this paper, we measured the ratio *Q* = *h*/*L* between the height *h* required to image the apical surface and the image size *L*, which provides a non-dimensional estimate of curvature. We find that *Q* is about 1.5% in our notum images, 3.5% in our retina images, 4.3% in our germband images, and 3.5% in our quail images, ensuring that the apical surfaces of tissues analyzed throughout the paper were reasonably flat.

### Flies and quails

For the experiments in the *Drosophila* notum, Ecad:GFP/Sqh:MCherry flies were used.

For the experiments in the *Drosophila* ommatidia, E-CAD:GFP; N-CAD:mkate2 flies were used^27^. Mosaic experiments were also described in a previous paper^27^.

For the experiments in the *Drosophila* germband, a; E-cad::GFP^KI^ fly line was used as wild type, embryos from a; tor^4^, E-cad::GFP^KIn^ were used as Torso−/− and dsRNAs against even-skipped injected in embryos form; E-cad::GFP^KI^ flies as previously described^2,6^ to obtain eve loss-of-function embryos.

For quail embryo stainings, quail embryos were fixed in ice cold 4% formaldehyde/PBS for at least 1 h, permeabilized in PBS/0.1% Triton X-100 (PBT 0.1%) before a blocking step in PBT 0.1%/2% BSA (from Roche)/10% FBS (from Gibco). Primary antibodies used in this study are mouse anti-ZO1 (Invitrogen ZO1-1A12), rabbit anti-pMyosin light chain 2 (Cell Signaling Technology CST-3671S and CST-3674S), mouse anti-β-Catenin (BD Transduction Laboratories™, clone 14) and rabbit anti-h/mCaspase3 (RD Systems AF835). Secondary antibodies coupled to AlexaFluor 488, 555, or 647 were obtained from Invitrogen and used at 1:200 dilutions. Embryos were then mounted with DAPI-containing Fluoromount-GTM (eBioscience) between slide and coverslip.

### Laser ablations

Laser ablation experiments in *Drosophila* were performed on a previously described setup^3^. Junction cuts in the retina were described in a previous article^27^. Line cuts in the germband were also described in a previous article^6^.

Laser ablation experiments in quail embryos were performed live using a 355-nm pulsed laser (75–100% power), a UGA-42 module from Rapp Optoelectronic coupled to a Zeiss LSM 880 and a 5X or 10X objective (see^31^ for details).

### Ablation measurements

In the *Drosophila* notum, we used kymographs along lines parallel to the severed junctions to automatically track the movement of vertices. Kymographs were then oversampled and treated with a Gaussian filter to avoid pixelation effects in vertex detection. Vertices positions were determined at each time point with a Gaussian fit of the intensity. Despite these efforts, the data can still be quite noisy. Besides, we needed to fit separately each single opening curve, without the possibility to average over several junctions as we wanted to do single junction comparisons with force inference. Thus, to determine the initial recoil velocity we had to perform a linear fit on the first 5 seconds. The fitting time was determined empirically, as too short fitting times are very much affected by noise, and too long fitting times yield poor estimates as opening is rather exponential or bi-exponential than linear. Note that we used linear fits of the onset of relaxation rather than exponential of bi-exponential fits of the full relaxation process for an empirical reason. Only fitting the first few points clearly focuses error minimization on the onset of relaxation, whereas fitting the whole relaxation with exponentials might overall give a better fit, but possibly at the expense of the onset of opening, as it is only a small subset of the relaxation curve.

In the *Drosophila* retina, automated detection with kymographs could not be used, due to smaller cells, edge curvatures, and higher signal loss following ablations. Hence, we used a manual tracking approach of the vertices, using FIJI. In this case, opening curves could be averaged over several junctions, which yielded much less noisy curves. Hence we could estimate the opening velocity on a much smaller timescale, looking at the first 250 ms. Note that the gradation observed is still found if we fit curves independently on a longer timescale (as it is done in the notum), then average velocities for each junction type. This strategy was actually the one used in our previous paper^27^, and yielded a similar gradation. Velocities determined here are closer to the actual “initial” velocity, as they are measured on a shorter timescale after the cut. The higher values found here suggest that it is indeed the case.

In the *Drosophila* germband, the opening velocities were determined by Particle Image Velocimetry (PIV), as several junctions are involved in the opening process. The measurement routine was described in a previous article^6^. In short, PIV is computed between a snapshot taken upon ablation and a snapshot taken 2 s after ablation. The velocity field is averaged in a region adjacent to the cut line to obtain a scalar velocity value.

In the quail embryo, tissue strain was evaluated based on the deformation of the tissue 2 minutes after the cut, from a PIV analysis of the resulting time-lapse movies. Note that the initial opening velocity could not be measured since relaxation occurred on a time scale comparable to the time taken to make the cuts (see^31^ for details). This prevented a quantitative analysis of initial recoil velocities vs. inferred stress.

### Statistics

We use Pearson’s correlation coefficient to determine the correlation in scatter plots. We use unpaired Student t-tests to determine whether distribution means are significantly different. N.S. stands for non-significant and is used when p > 0.05. If p < 0.05, its value is reported directly on the graph. Error bars on all plots represent the standard error of the mean.

## Supplementary information

Supplementary Figures

## Data Availability

Data will be made available upon request.
