# Supplementary material for: Experimental validation of force inference in epithelia from cell to tissue scale
Source: Sci Rep. 2019 Oct 10;9:14647. doi: 10.1038/s41598-019-50690-3 (PMC6787039; doi:10.1038/s41598-019-50690-3)
Supplement: Supplementary file 1 — Supplementary Figures [file 41598_2019_50690_MOESM1_ESM.pdf]

Experimental validation of force inference in epithelia from cell to tissue scale

W. Kong, O. Loison, P. Shivakumar, E. Chan, M. Saadaoui, C. Collinet, P.F Lenne, R. Clément

SUPPLEMENTARY FIGURES

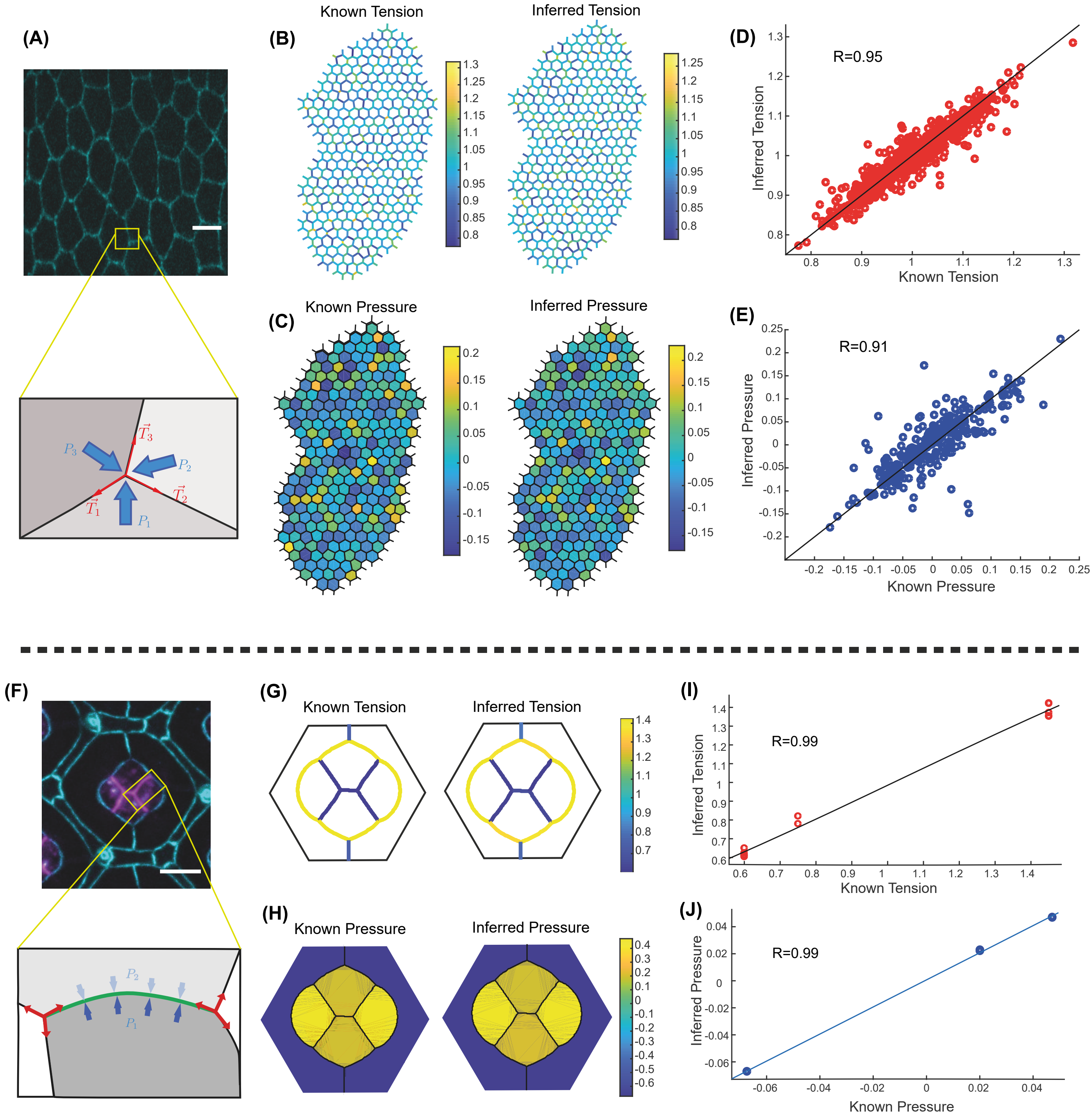

Supplementary Figure S1 - Validation of force inference on synthetic data
(A) Scheme of the force balance in the Bayesian force inference method. Tensions and pressures act as forces directly applied to vertices, and the force balance is written at each vertex. Sample image: Drosophila notum around 22h after pupa formation. Scale bar: 5µm. (B) Known (left) vs. inferred (right) tension map in a tissue simulated with Surface Evolver (246 cells, 873 edges). (C) Known (left) vs. inferred (right) pressure map. (D) Inferred tensions vs. known tensions. (E) Inferred pressures vs. known pressures. (F) Scheme of the force balance in the Laplace force inference method. Tensions act as forces applied to vertices tangent to the edges, and pressures are related to edge tension and curvature by Laplace's law. Sample image: Drosophila ommatidium 41h after pupa formation. Scale bar: 5µm. (G) Known (left) vs inferred (right) tension map in an ommatidium simulated with Surface Evolver. (H) Known (left) vs inferred (right) pressure map. (I) Inferred tensions vs. known tensions. (J) Inferred pressures vs. known pressures.

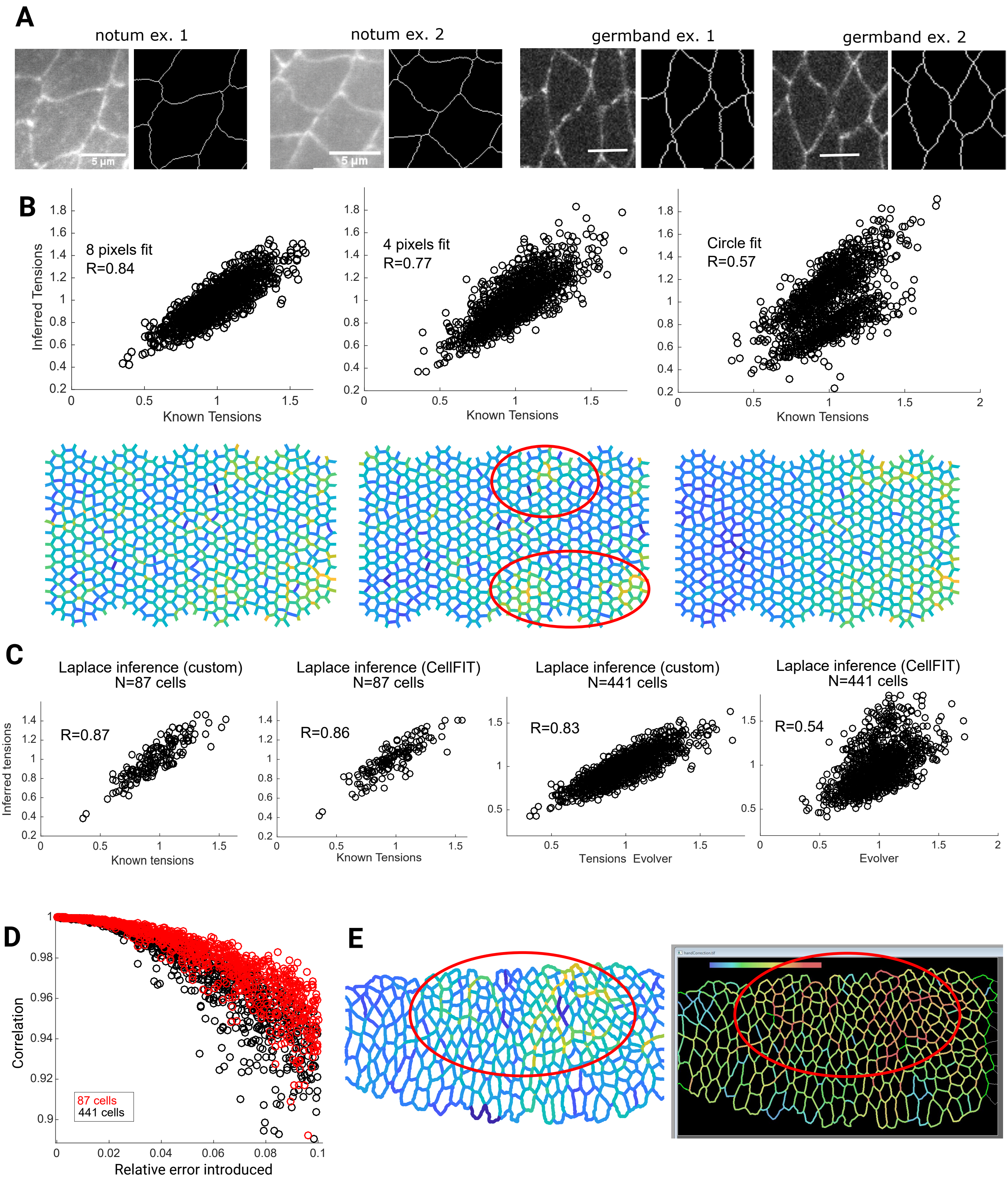

Supplementary Figure S2 - Source of errors in tangent determination

(A) Examples of edges with the shape of an open S. (B) Correlation plots (R=Pearson's coefficient) and inference maps obtained with our Laplace inference implementation with 8 pixels (left), 4 pixels (middle), or circle fit (right) determination of the tangent. Red circles show the regions where errors arise. (C) Correlations obtained with Laplace inference (custom or CellFIT) for 2 tissue sizes. (D) Correlation between inference with and without introduced errors vs. relative error amplitude. Introduced errors affect only one element of the matrix in each iteration. (E) Example of error propagation in the germband of Drosophila, with our implementation (left) and CellFIT (right). High tension values emerge in the same regions.

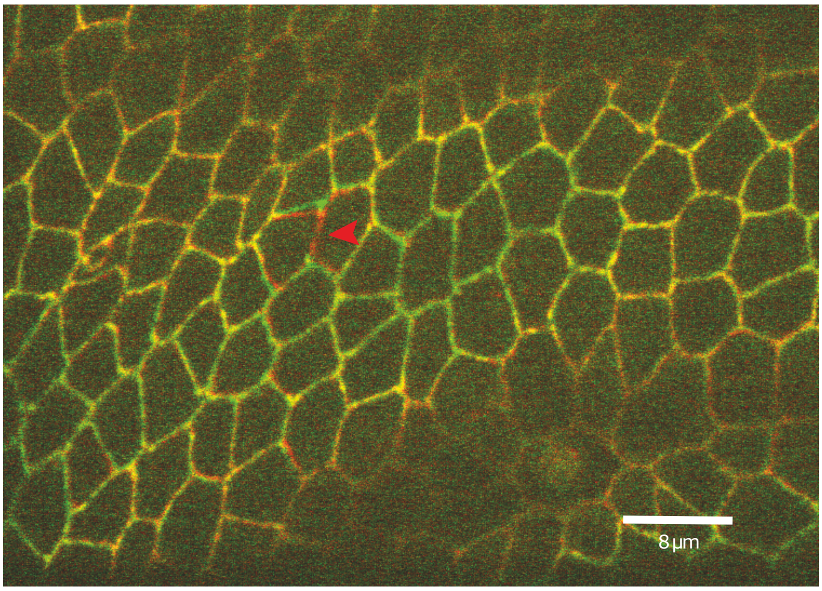

Pre-ablation Post-ablation

Supplementary Figure S3 - Impact of point ablation on surrounding tissue geometry
Overlay of the tissue geometry prior (red) and after (green) cutting a single junction (marked by an arrowhead). The displacement in the surrounding cells decays rapidly, and is hardly noticeable above a one-cell distance.

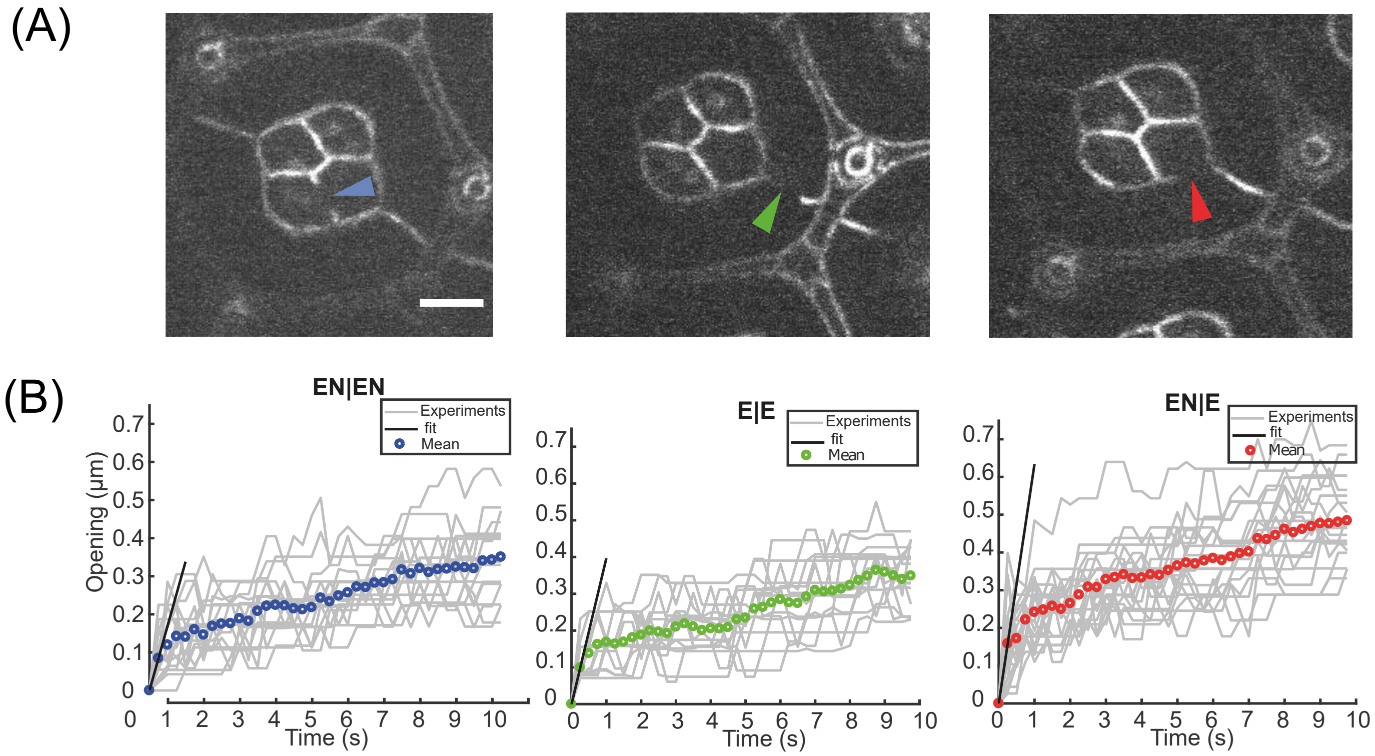

Supplementary Figure S4 - Ablations in the ommatidia

(A) Snapshots of ablation experiments performed for each type of junction. (B) Averaged opening dynamics for each type of junction (EN|EN: N=19, E|E: N=16, EN|E: N=22). Individual opening curves are shown in light gray.

(A)

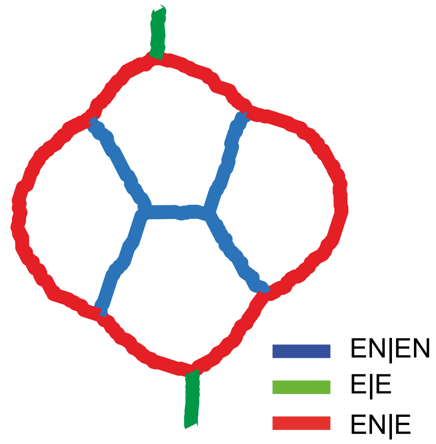

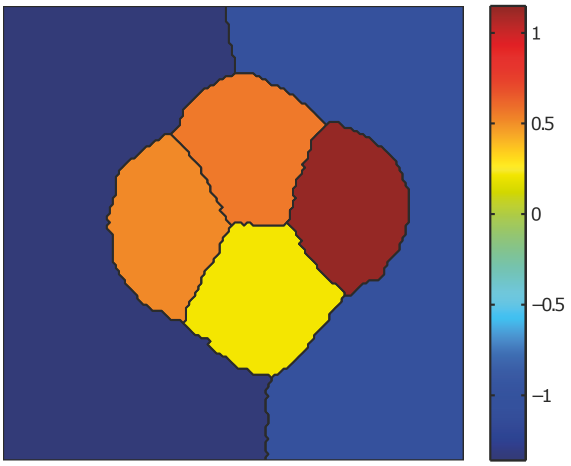

(B)

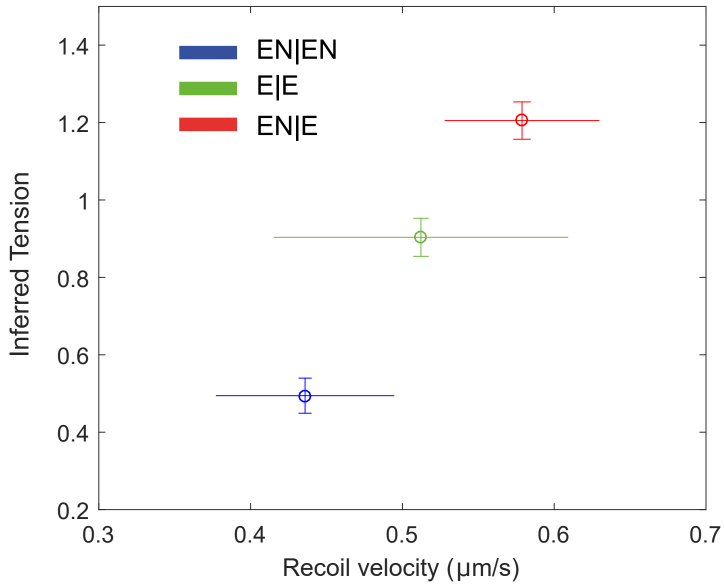

Supplementary Figure S5
(A) Left: Junction types. Right: Pressure map obtained using Laplace force inference in the averaged WT ommatidium.
(B) Inferred tensions vs. recoil velocity averaged over all 5 mutant configurations (N=5).

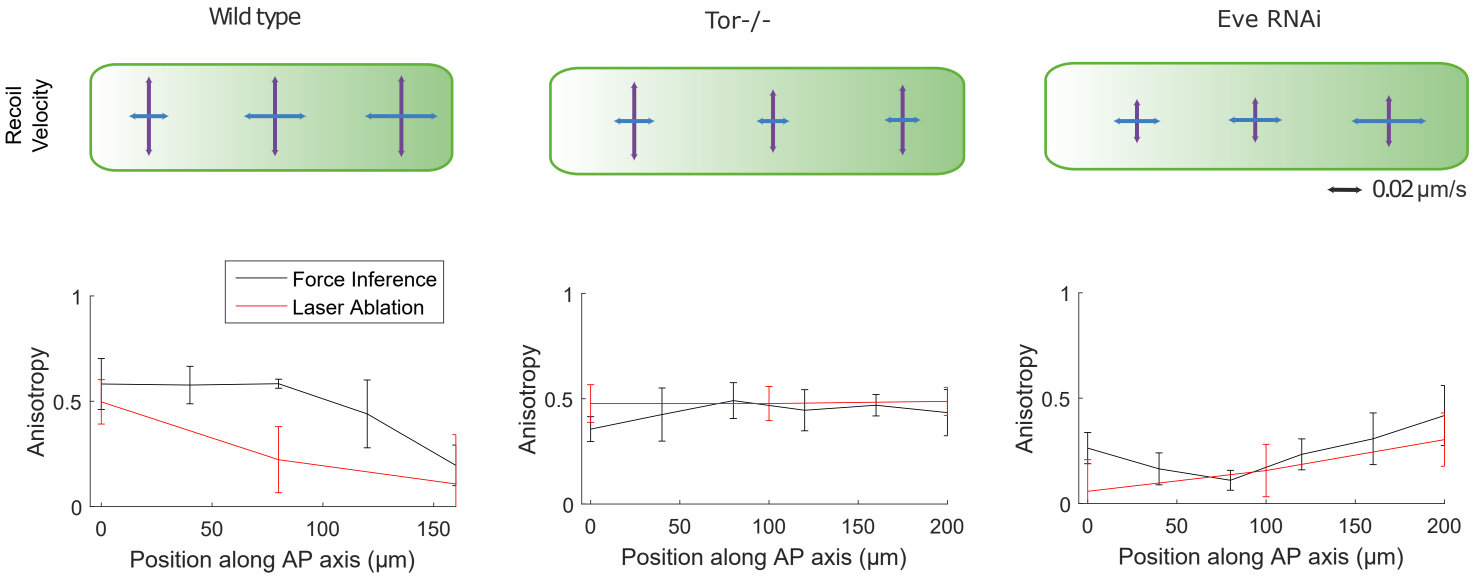

Supplementary Figure S6 - Stress anisotropy along the AP axis
Top : Horizontal and vertical recoil velocities measured by laser ablation in different conditions.
Bottom : Anisotropy along the AP axis measured by force inference or laser ablation in different conditions. Anisotropy is defined as A=1-m/M, where M is the amplitude in the principal direction, and m the amplitude in the other direction.

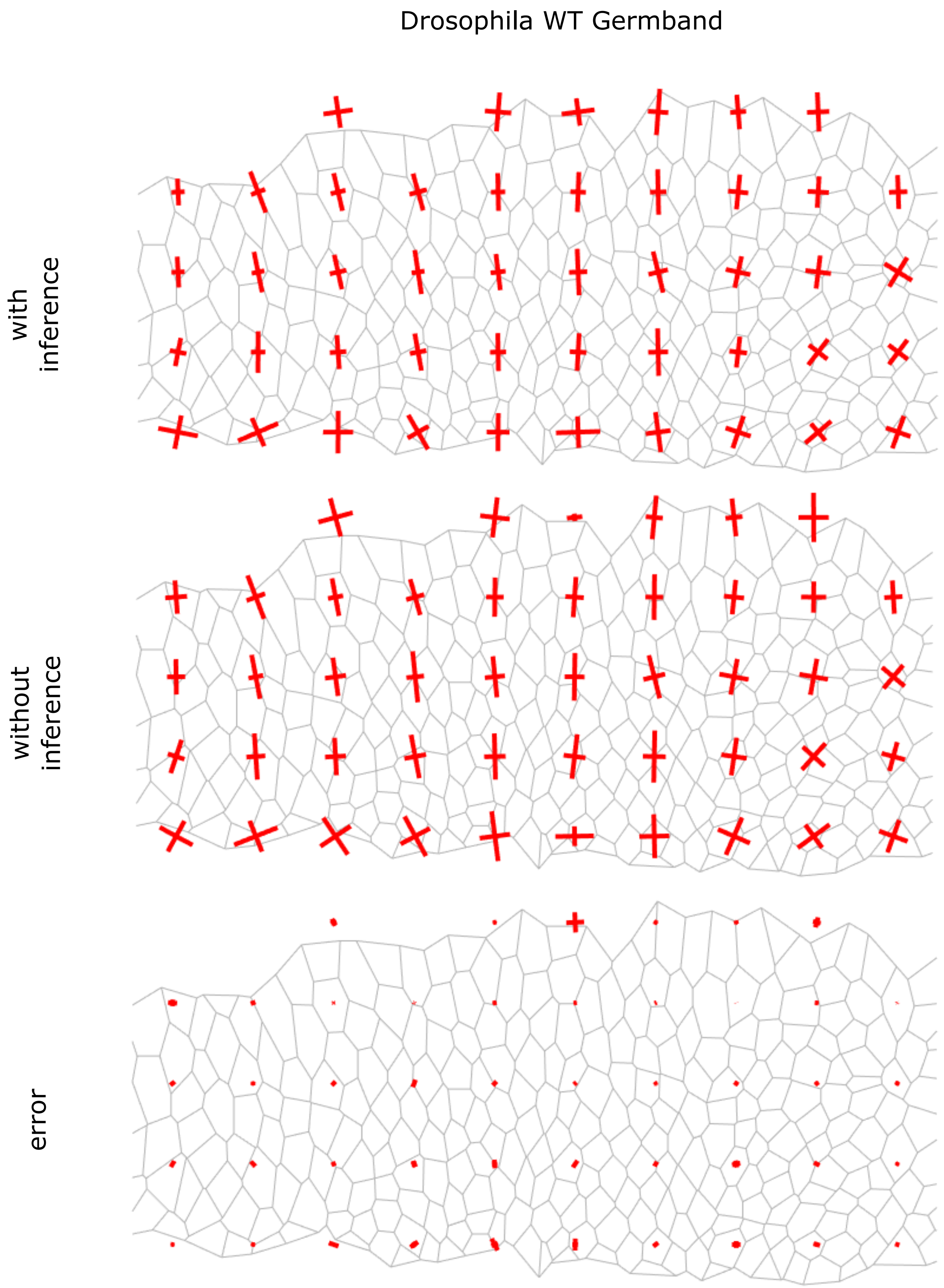

Supplementary Figure S7 - Stress estimates from cell shapes only in the WT germband
Top - Stress computed using Batchelor formula and the results of force inference
Middle - Stress computed using Batchelor formula assuming that tensions and pressures are homogeneous
Bottom - Error (top minus middle)

Quail Embryo

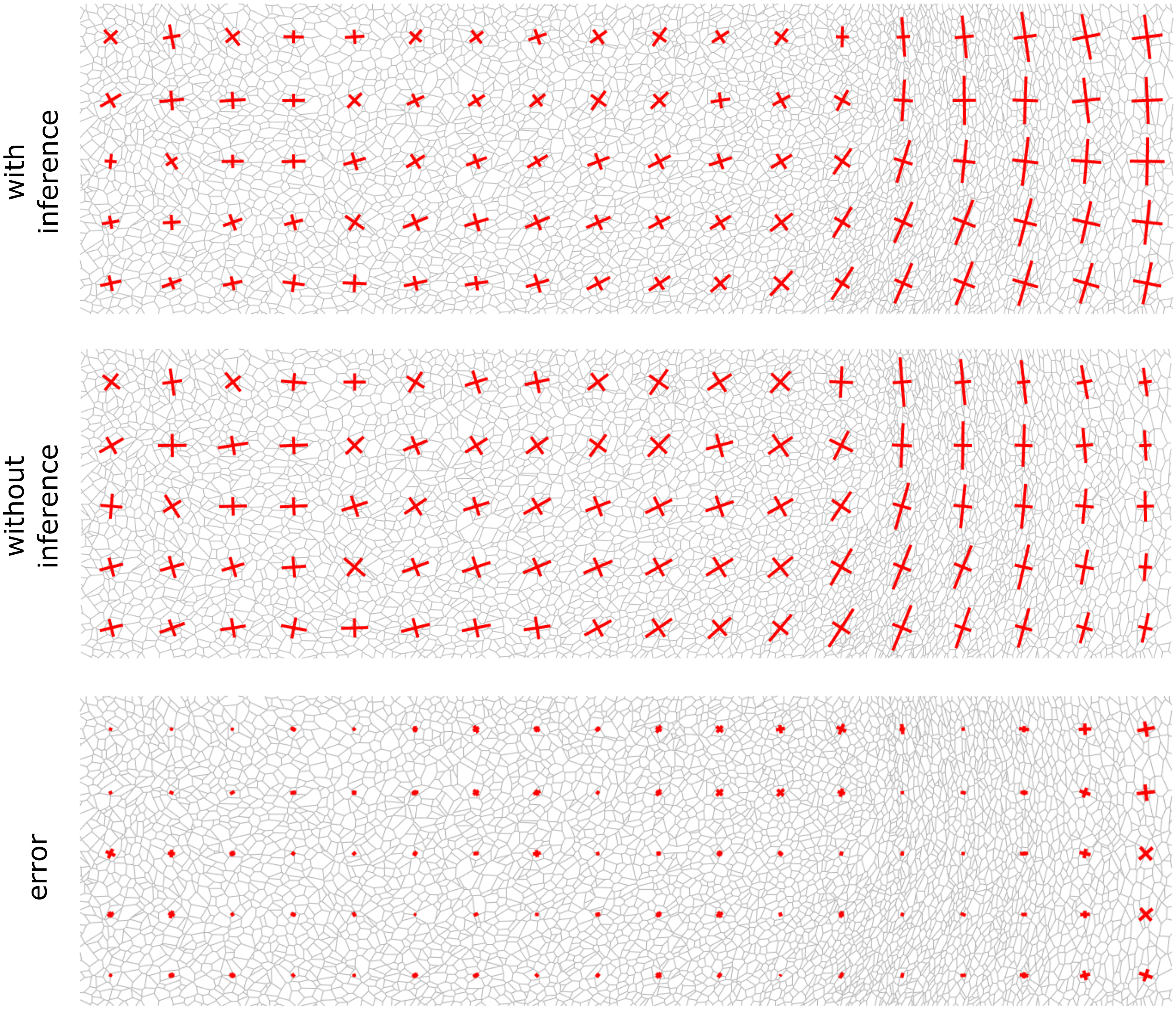

Supplementary Figure S8 - Stress estimates from cell shapes only in the quail embryo
Top - Stress computed using Batchelor formula and the results of force inference
Middle - Stress computed using Batchelor formula assuming that tensions and pressures are homogeneous
Bottom - Error (top minus middle)
